# Methylprednisolone up-regulates annexin A1 (ANXA1) to inhibit the inflammation, apoptosis and oxidative stress of cigarette smoke extract (CSE)-induced bronchial epithelial cells, a chronic obstructive pulmonary disease in vitro model, through the formyl peptide receptor 2 (FPR2) receptors and the adenosine 5’-monophosphate (AMP)-activated protein kinase (AMPK) pathway

**DOI:** 10.1080/21655979.2022.2031769

**Published:** 2022-02-06

**Authors:** Chan Yu, Linghui Zhang

**Affiliations:** a The First Department of Respiratory Medicine, Chengdu Eighth People’s Hospital (Geriatric Hospital of Chengdu Medical College), Chengdu City, China; b Department of Internal Medicine, Department of Clinical Medicine, Shijiazhuang Medical College, Shijiazhuang City, China

**Keywords:** Methylprednisolone, bronchial epithelial cells, ANXA1, chronic obstructive pulmonary disease, FPR2/AMPK pathway

## Abstract

Chronic obstructive pulmonary disease (COPD) is a progressive degenerative disease, of which smoking is the main causer. We carried out this study with the aim of exploring the underlying mechanism of methylprednisolone (MP) treating the COPD. To stimulate COPD in vitro, cigarette smoke extract (CSE)was employed to induce human bronchial epithelial cells BEAS-2B. With the help of MTT and Tunel assays, the viability and apoptosis of BEAS-2B cells after indicated treatment were assessed. The levels of inflammatory response and oxidative stress were determined by the changes of markers basing on their commercial kits. Additionally, annexin A1 (ANXA1) expressions at both protein and mRNA levels were assessed with Western blot and Reverse transcription‑quantitative PCR (RT-qPCR). Moreover, the expressions of apoptosis- and formyl peptide receptor 2 (FPR2) receptors and the adenosine 5’-monophosphate (AMP)-activated protein kinase (AMPK) pathway-related proteins were determined with Western blot., related proteins and proteins. As a result, MP up-regulated the ANXA1 expression in CSE-induced BEAS-2B cells. MP enhanced the viability but suppressed the apoptosis, inflammatory response and oxidative stress of CSE-induced BEAS-2B cells via regulating FPR2/AMPK pathway, while ANXA1 knockdown exhibited oppositive effects on them. In conclusion, MP up-regulated ANXA1 to inhibit the inflammation, apoptosis and oxidative stress of BEAS-2B cells induced by CSE, alleviating COPD through suppressing the FPR2/AMPK pathway.

## Introduction

Chronic obstructive pulmonary disease (COPD) has gained worldwide attention in recent years. Patients mainly show dyspnea and obvious symptoms of upper and lower respiratory tract [1,2]. According to epidemiological survey, the percentage of people over 40 years old diagnosed with COPD in China is up to 13.7 and this trend is still on the rise. Currently, there are about 100 million COPD patients in China [3]. Smoking is the main pathological factor that causes airway wall inflammation and injury, and most cases of COPD are caused by smoking [4]. The improved symptoms, pulmonary function and arterial blood gas analysis could be observed in COPD patients in acute exacerbation period after intravenously administrated with methylprednisolone (MP) [5]. Single dose of MP injection was used to treat patients with asthma and COPD for more than 1 month without significant side effects demonstrated a safe strategy for them [6]. However, the mechanism of MP in the treatment of COPD hasn’t been elucidated clearly.

MP has a wide range of anti-inflammatory effects. Intravenous injection of MP could rapidly ameliorate noninfectious uveitis [7]. Being an immunosuppressive therapy, MP exserted desirable effects on reducing respiratory inflammation in patients with COVID-19 [8]. MP alleviated the persistent post-ischemic inflammatory response in hypoxic-ischemia model of perinatal stroke rats [9]. MP had anti-inflammatory and antioxidant effects on L-arginine-induced acute pancreatitis [10]. MP sodium succinate reduced blood-brain barrier destruction and inflammation in intracranial hemorrhage model mice [11]. Drugbank (https://go.drugbank.com/) predicts that Annexin A1 (ANXA1) is a target for MP.

ANXA1, which contains 12 introns and 13 exons, belongs to calcium-ion dependent phospholipid-binding protein family [12]. MP mediated by spinal ANXA1 alleviated the writhing response and spinal PGE2 level in ANXA1+/+ mice, and thus played an anti-nociceptive role within the spinal cord [13]. Han et al. held the opinion that the reduction of inflammatory cytokines mediated by ANXA1 could promote the subsidence of inflammation in lung injury [14]. ANXA1 mimetic peptide Ac2-26 alleviated kidney injuries in db/db mice by suppressing the inflammatory state [15]. Methanolic extract of Cariniana rubra (MECr) inhibited inflammation by promoting the ANXA1 expression and inhibiting the TNF-α and leukocyte migration in carrageenan air pouch inflammation model [16]. ANXA1 mimetic peptide Ac2-26 alleviated the inflammation in COPD via reducing the levels of pro-inflammatory mediators and increasing anti-inflammatory cytokines [17]. Piperlongumine attenuated inflammation in cigarette smoke induced COPD mice model partially by promoting the expression of ANXA1 [18]. Normally, the existance of ANXA1 can be mainly found in the cytosol. When stimulated by inflammatory cytokines, ANXA1 can move to the cell surface in large quantities and inhibit the migration of white blood cells to the inflammatory site, thus having an anti-inflammatory effect [19].

ANXA1 alleviated inflammation in mice with meningitis and neuroinflammation in mice with intracerebral hemorrhage by regulating FPR2/p38/COX-2 pathway [20,21]. ANXA1 promoted proliferation and migration of Schwann cells to accelerate nerve regeneration via the activation of formyl peptide receptor 2 (FPR2) receptors and the adenosine 5’-monophosphate (AMP)-activated protein kinase (AMPK) pathway [22]. Whether ANXA1 regulated the FPR2/AMPK pathway in COPD needs to be investigated.

In the present study, we performed the cigarette smoke extract (CSE)-induced bronchial epithelial cells, a chronic obstructive pulmonary disease in vitro model and to investigate the role of MP in apoptosis, inflammatory response as well as oxidative stress of CSE-induced bronchial epithelial cells. Then, ANXA1 expression in CSE-induced bronchial epithelial cells was determined and the effects of ANXA1 on MP treatment for the apoptosis, inflammatory response as well as oxidative stress in CSE-induced bronchial epithelial cells were analyzed. Lastly, the potential action of the detailed molecular mechanism was explored.

## Materials and Methods

### Cell culture and treatment

Procell Life Science & Technology Co., Ltd (Wuhan, China) was the provider of human bronchial epithelial cells BEAS-2B (cat. no. CM-0496). The RPMI 1640 medium (Gibco) which contained 10% fetal bovine serum (FBS), 100 U/mL penicillin and 100 U/mL streptomycin was employed to foster these cells with 5% CO_2_ at 37°C.

Being exposure to cigarette smoke extract (CSE) 1, 2, 5 and 10%, BEAS-2B cells were cultured in RPMI1640 medium without FBS for 24 h.

After the pretreatment with 2, 5 and 10 μM methylprednisolone (MP) for 2 h, BEAS-2B cells were then administrated with 5% CSE for 24 h.

### CSE preparation

The preparation of CSE was operated in line with the slightly modified previous method [23]. Cigarette smoke from commercial Marlboro Red cigarettes (0.8 mg of nicotine and 10 mg of tar) was drawn into a modified 50 mL syringe apparatus. After complete smoke of cigarette, 100% CSE was obtained by mixing the smoke with 20 mL serum-free RPMI 1640 through vigorous shaking. In order to clear out large particles and bacteria, a 0.22 μm filter was applied to filter the solution. Finally, 100% CSE was diluted with RPMI 1640 to the required concentration and induced cells within 15 min.

### Cell transfection

GenePharma (Shanghai, China) was the provider of the transfection plasmids. BEAS-2B cells were inoculated into 6-well plates, after which was the transfection with si-NC, si-ANXA1#1 and si-ANXA1#2 under the help of Lipofectamine 2000 at 70% of confluence. At 48 h, the transfected cells were harvested for verification of the transfection efficacy and subsequent experiments.

### MTT assay

Inoculated into a 96-well plate, the cells were fostered at 37°C with 5% CO_2_ overnight. Administrated with MTT solution (final concentration 0.5 mg/ml), the cells were subsequently incubated for another 4 h. After that, the formazan was dissolved by 150 μl dimethyl sulfoxide (DMSO). The absorbance was detected at 570 nm with a Thermomax microplate reader.

### Tunel assay

With the application of Tunel assay (Millipore), the cell apoptosis was evaluated. After indicated treatment, the fixation of BEAS-2B cells with 4% paraformaldehyde was conducted for 1 h and the permeabilization with 0.1% Triton X-100 in PBS lasted 2 min. Thereafter, the cells were rinsed by PBS and then was the incubation with TUNEL reaction mixture. 0.1 µg/ml DAPI was also applied to incubate the cells at 30°C for 30 min. The observation of cell apoptosis was captured by a fluorescence microscope (IX73; Olympus) (magnification, x200).

### Western blot analysis

Total proteins extracted from lysates of BEAS-2B cells in each group were prepared in RIPA lysis buffer. With the help of BCA method, the protein concentration was assessed. The separation of the cells with 10% SDS-PAGE was performed and then was the transferring of proteins onto PVDF membranes (Millipore). After inhibition with 5% skim milk, primary antibodies against Bcl-2, Bax, cleaved caspase 3, cleaved PARP, ANXA1, FPR2, p-AMPK, AMPK and GAPDH were adopted to culture PVDF membranes at 4°C overnight. Next day, the co-incubation of HRP-conjugated secondary antibodies and membranes was conducted. The enhanced chemiluminescence (ECL; Santa Cruz Biotechnology) detection was used to show the protein bands and density of bands were analyzed by Image J 1.8.0 (National Institute of Health).

### Determination of the levels of inflammatory response and oxidative stress

Tumor necrosis factor α (TNF-α), interleukin 6 (IL-6) and IL-1β levels in the BEAS-2B cells from each group were detected by commercial ELISA kits of TNF-α (cat. no. PT518), IL-6 (cat. no. PI330) and IL-1β (cat. no. PI305) (all from Beyotime). Superoxide dismutase (SOD) (cat. no. E-BC-K020-M), glutathione peroxidase (GSH-Px) (cat. no. E-BC-K096-S) and malondialdehyde (MDA) (cat. no. E-BC-K028-M) kits (all from Elabscience) were used to determine the levels of SOD, GSH-Px and MDA.

### Reverse transcription‑quantitative PCR (RT-qPCR)

The RNA that isolated with TRIzol reagent (Invitrogen, Carlsbad) was reversely transcribed into cDNAs with the application of PrimeScript RT Reagent (TaKaRa). The subjection of obtained cDNA to qRT-PCR was operated by SYBR®Premix Ex Taq™ (TaKaRa).U6 was viewed to be an internal reference. With the favor of 2^−ΔΔCq^ method [24], the level of gene was evaluated. The following were the primers we used in our study: ANXA1 forward, 5′-TGATGAACTTCGTGCTG-3′ and reverse, 5′-TGGTTTGCTTGTGGC-3′; GAPDH forward, 5′-AGAAGGCTGG GGCTCATTTG-3′ and reverse, 5′-AGGGGCCATC CACAGTCTTC-3′.

### Statistical Analysis

The results that obtained from our experiments were displayed as mean values ± standard deviation (SD). GraphPad Prism 8.0 was employed with the aim of analyzing the data, and multiple comparison test was basing on one-way ANOVA with Tukey’s test. P lower than 0.05 was believed to be of statistical significance.

## Results

### MP increases the viability of CSE-induced BEAS-2B cells

**Figure 1.**
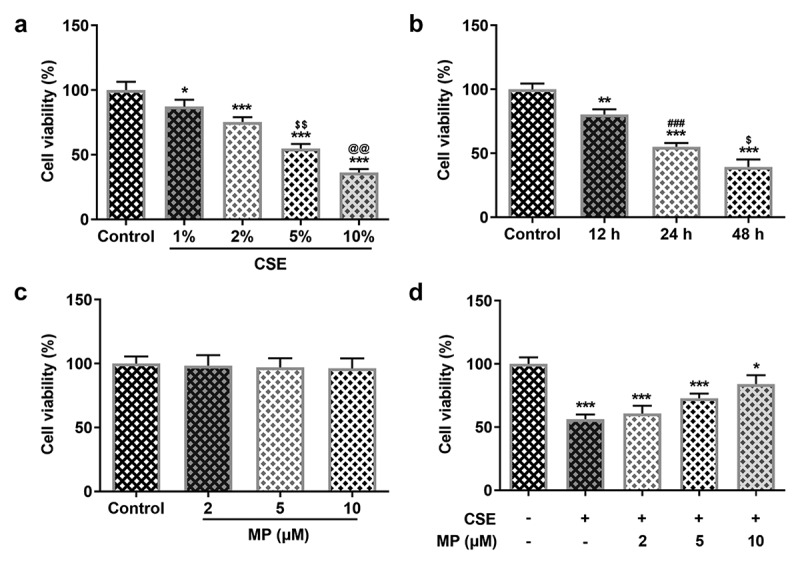
MP increases the viability of CSE-induced BEAS-2B cells. (a) The viability of BEAS-2B cells treated with 1, 2, 5 and 10% CSE for 24 h was detected by CCK-8 assay. *P < 0.05 and ***P < 0.001 vs. Control group. ^$$^P < 0.01 vs. 2% CSE group. ^@@^P < 0.01 vs. 5% CSE group. (b) The viability of BEAS-2B cells treated with 5% CSE for 12, 24 and 48 h was detected by CCK-8 assay. **P < 0.01 and ***P < 0.001 vs. Control group. ^###^P < 0.001 vs. 12 h group. ^$^P < 0.05 vs. 24 h group. (c) The viability of BEAS-2B cells treated with 2, 5 and 10 μM MP was detected by CCK-8 assay. (d) The viability of BEAS-2B cells treated MP and CSE was detected by CCK-8 assay. *P < 0.05 and ***P < 0.001 vs. Control group.

To determine the concentrations and application time of CSE on BEAS-2B cells, cell viability detected by MTT assay was conducted. At the beginning, we initially administrated the cells with different concentrations (1, 2, 5 and 10%) CSE and employed MTT to evaluated the viability. Compared with the Control, the viability of BEAS-2B cells was significantly reduced after CSE induction (Figure 1(a)). It was noted that CSE induction suppressed the cell viability in a dose-dependent manner. In our study, we chose 5% CSE for the subsequent experiments. As Figure 1(b) demonstrated, the viability of 5% CSE-induced BEAS-2B cells was gradually decreased as time passed by, revealing that CSE exhibited inhibitory effects on cell viability also in a time-dependent manner. Therefore, BEAS-2B cells induced by 5% CSE for 24 h was used for the COPD in vitro model. To investigate the different concentrations of MP on BEAS-2B cells or CSE-induced BEAS-2B cells, MTT assay was used to detect the cell viability. When MP with different concentrations (2, 5 and 10 μM) was applied to administrate BEAS-2B cells, the cell viability stayed unchanged(Figure 1(c)). Nevertheless, the pretreatment of MP (2, 5 and 10 μM) could greatly revived the viability of CSE induced BEAS-2B cells in comparison with that in CSE group (Figure 1(d)).

### MP inhibits the apoptosis of CSE-induced BEAS-2B cells

**Figure 2.**
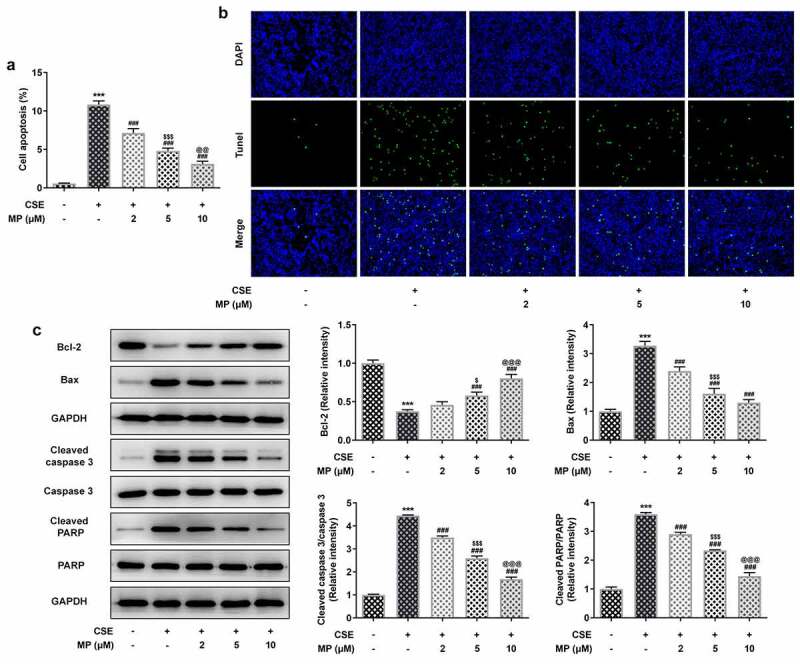
MP inhibits the apoptosis of CSE-induced BEAS-2B cells. (a and b) The apoptosis of BEAS-2B cells treated MP and CSE was detected by Tunel assay. (c) The expression of apoptosis-related proteins in CSE-induced BEAS-2B cells pre-treated with MP was determined by Western blot. ***P < 0.001 vs. Control group. ^###^P < 0.001 vs. CSE group. ^$^P < 0.05 and ^$$$^P < 0.001 vs. 2 μM + CSE group. ^@@^P < 0.01 and ^@@@^P < 0.001 vs. 5 μM + CSE group.

The apoptosis and related protein expression in CSE-induced BEAS-2B cells after MP treatment were determined by Tunel assay and Western blot. The apoptosis of CSE-induced BEAS-2B cells was gradually decreased after the pre-treatment of 2, 5 and 10 μM MP (Figures 2(a,b)). Additionally, it was noted that MP treatment upregulated Bcl-2 expression but downregulated the expressions of Bax, cleaved caspase 3 and cleaved PARP in CSE-induced BEAS-2B cells in contrast with the CSE group (Figure 2(c)).

### MP attenuates the inflammatory response and oxidative stress of CSE-induced BEAS-2B cells

**Figure 3.**
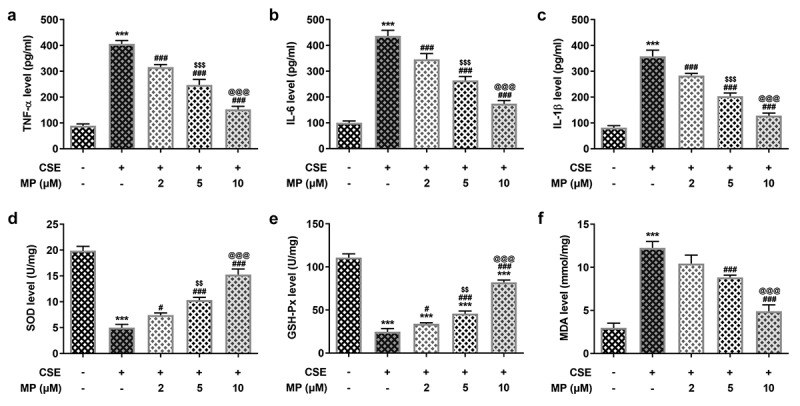
MP attenuates the inflammatory response and oxidative stress of CSE-induced BEAS-2B cells. The levels of inflammatory factors TNF-α (a), IL-6 (b) and IL-1β (c) in CSE-induced BEAS-2B cells pre-treated with MP were determined by ELISA assay kits. The levels of oxidative stress factors SOD (d), GSH-Px (e) and MDA (f) in CSE-induced BEAS-2B cells pre-treated with MP were determined by assay kits. ***P < 0.001 vs. Control group. ^#^P < 0.05, ^#^P < 0.01 and ^###^P < 0.001 vs. CSE group. ^$$^P < 0.01 and ^$$$^P < 0.001 vs. 2 μM + CSE group. ^@@@^P < 0.001 vs. 5 μM + CSE group.

The inflammatory response and oxidative stress in CSE-induced BEAS-2B cells after MP treatment were determined. TNF-α, IL-6 and IL-1β are inflammatory factors reflecting the inflammatory response and SOD, GSH-Px and MDA are oxidative stress factors reflecting the oxidative stress. As Figures 3(a,b) vividly depicted, MP suppressed the levels of TNF-α, IL-6, IL-1β and MDA while promoted the levels of SOD and GSH-Px in CSE-induced BEAS-2B cells.

### MP upregulates the ANXA1 expression in CSE-induced BEAS-2B cells and knockdown of ANXA1 promotes the apoptosis of CSE-induced BEAS-2B cells treated with MP

**Figure 4.**
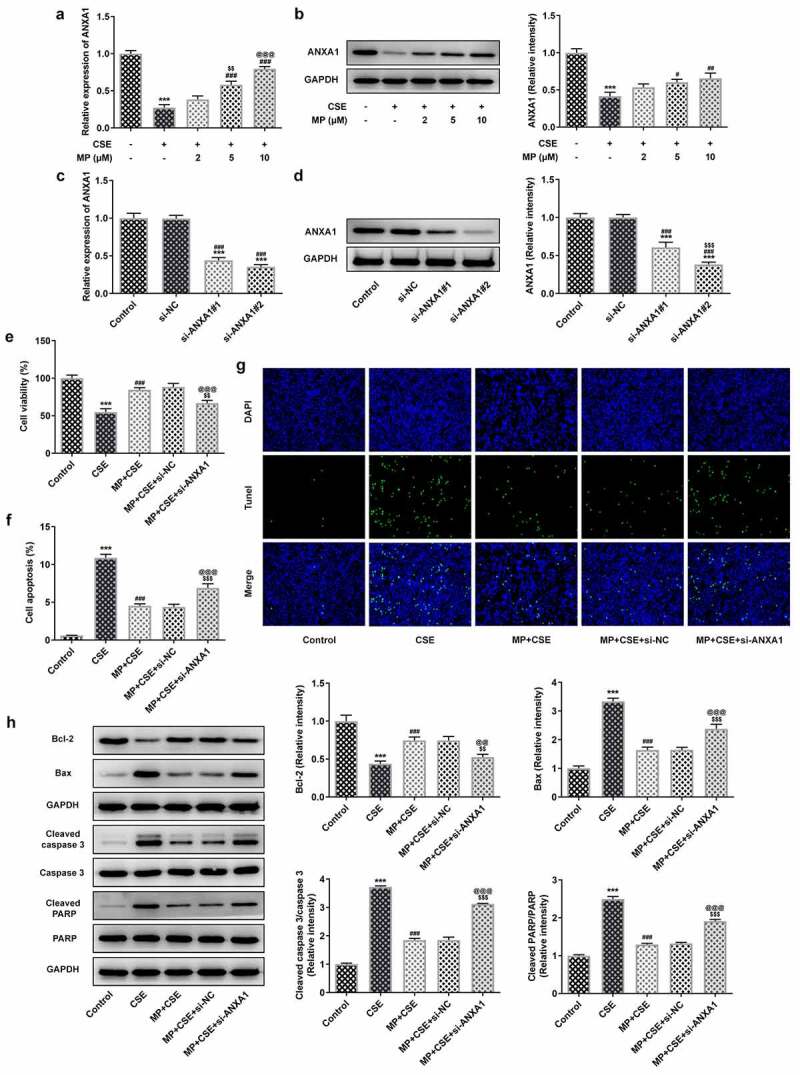
MP upregulates the ANXA1 expression in CSE-induced BEAS-2B cells and knockdown of ANXA1 promotes the apoptosis of CSE-induced BEAS-2B cells treated with MP. mRNA (a) and protein (b) expression of ANXA1 in CSE-induced BEAS-2B cells treated with MP were respectively detected by RT-qPCR and Western blot. ***P < 0.001 vs. Control group. ^#^P < 0.05, ^#^P < 0.01 and ^###^P < 0.001 vs. CSE group. ^$$^P < 0.01 vs. 2 μM + CSE group. ^@@@^P < 0.001 vs. 5 μM + CSE group. mRNA (c) and protein (d) expression of ANXA1 in BEAS-2B cells transfected with si-ANXA1#1/2 were respectively detected by RT-qPCR and Western blot. ***P < 0.001 vs. Control group. ^###^P < 0.001 vs. si-NC group. ^$$$^P < 0.001 vs. si-ANXA1#1 group. (e) The viability of MP+CSE-induced BEAS-2B cells transfected si-ANXA1 was detected by CCK-8 assay. (f and g) The apoptosis of MP+CSE-induced BEAS-2B cells transfected si-ANXA1 was detected by Tunel assay. (h) The expression of apoptosis-related proteins in MP+CSE-induced BEAS-2B cells transfected si-ANXA1 was determined by Western blot. ***P < 0.001 vs. Control group. ^###^P < 0.001 vs. CSE group. ^$$^P < 0.01 and ^$$$^P < 0.001 vs. MP + CSE group. ^@@^P < 0.01 and ^@@@^P < 0.001 vs. MP + CSE + si-NC group.

The mRNA and protein expression of ANXA1 in CSE-induced BEAS-2B cells with or without MP treatment were detected by RT-qPCR analysis and Western blot. The mRNA and protein expression of ANXA1 were reduced in CSE-induced BEAS-2B cells and upregulated when BEAS-2B cells were pretreated with MP (Figures 4(a,b)). The mRNA and protein expression of ANXA1 in BEAS-2B cells were determined to confirm the transfection effect. The mRNA and protein expression of ANXA1 were downregulated in BEAS-2B cells transfected with si-ANXA1#1/2 compared with that in control and si-NC groups, and si-ANXA1#2 was chosen for the next experiment (Figures 4(c,d)). To see whether ANXA1 knockdown weakened the effect of MP on CSE-induced BEAS-2B cells, the viability, apoptosis and related protein expression were detected. CSE reduced the BEAS-2B cells viability (Figure 4(e)) and promoted the apoptosis (Figures 4(f,g)), while MP pre-treatment imparted oppositive effects on them. Knockdown of ANXA1 suppressed the viability and enhanced the apoptosis of MP pre-treated and CSE induced BEAS-2B cells. Results in Figure 4(h) revealed that MP administration improved Bcl-2 expression but diminished the expressions of Bax, cleaved caspase 3 and cleaved PARP in CSE-induced BEAS-2B cells while ANXA1 knockdown reversed the effects of MP on these proteins, evidenced by the reduced Bcl-2 expression as well as enhanced expressions of Bax, cleaved caspase 3 and cleaved PARP in MP + CSE + si-ANXA1 group. decreased the expression of and increased the expression of The above changes could be reversed by MP and the role of MP in CSE-induced BEAS-2B cells was weaken by knockdown of ANXA1.

### Knockdown of ANXA1 enhances the inflammatory response and oxidative stress, and suppresses the FPR2/AMPK pathway of CSE-induced BEAS-2B cells treated with MP

**Figure 5.**
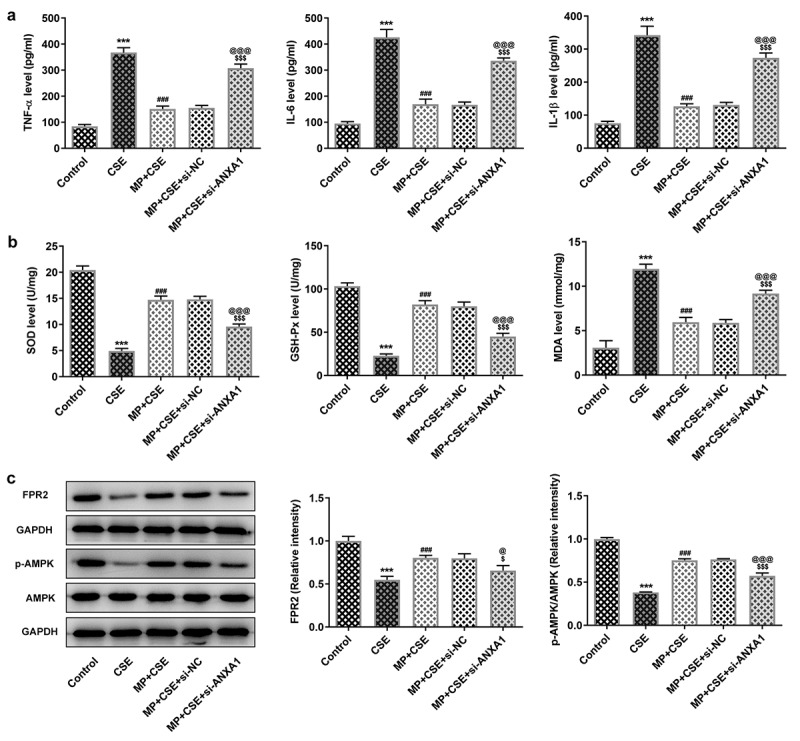
Knockdown of ANXA1 enhances the inflammatory response and oxidative stress, and suppresses the FPR2/AMPK pathway of CSE-induced BEAS-2B cells treated with MP. (a) The levels of inflammatory factors TNF-α, IL-6 and IL-1β in MP+CSE-induced BEAS-2B cells transfected si-ANXA1 were determined by ELISA assay kits. (b) The levels of oxidative stress factors SOD, GSH-Px and MDA in MP+CSE-induced BEAS-2B cells transfected si-ANXA1 were determined by assay kits. (c) The expression of FPR2/AMPK pathway in MP+CSE-induced BEAS-2B cells transfected si-ANXA1 was analyzed by Western blot. ***P < 0.001 vs. Control group. ^###^P < 0.001 vs. CSE group. ^$^P < 0.05 and ^$$$^P < 0.001 vs. MP + CSE group. ^@^P < 0.05 and ^@@@^P < 0.001 vs. MP + CSE + si-NC group.

To see whether ANXA1 knockdown weakened the effect of MP on CSE-induced BEAS-2B cells, the inflammatory response and oxidative stress were detected. According to Figure 5(a,b), CSE activated the inflammatory response and oxidative stress and MP suppressed the inflammatory response and oxidative stress in BEAS-2B cells caused by CSE. However, knockdown of ANXA1 weakened the inhibition effect of MP on the inflammatory response and oxidative stress in BEAS-2B cells caused by CSE. To see whether FPR2/AMPK pathway was related to the MP treatment for CSE-induced BEAS-2B cells, the expression of FPR2/AMPK pathway was detected. CSE suppressed the FPR2/AMPK pathway in BEAS-2B cells and MP activated the FPR2/AMPK pathway in CSE-induced BEAS-2B cells. However, knockdown of ANXA1 reversed the effect of MP on the FPR2/AMPK pathway in CSE-induced BEAS-2B cells (Figure 5(c)).

## Discussion

COPD, an obstructive lung disease with restricted airflow, contributes to an increasing rate of mortality year by year [25]. At present, the treatment plan of COPD mostly adopts the combination of drugs and smoking cessation and psychological treatment. In severe cases, oxygen inhalation and hormone therapy are required, lowering the quality of patient’s life [26,27].

For patients with COPD, the application value of glucocorticoids has been clinically confirmed. MP is a synthetic medium effective glucocorticoid, which has been widely used in the treatment of COPD in recent years [28–30]. The plasma free composition level of MP is high, with strong penetration in the lung tissue. It can be used in the treatment of COPD patients with rapid effect and significant anti-inflammatory effect. Intravenous infusion of prescription medication can also promote drug action on the whole body and reduce the body’s inflammatory reaction, to promote the remission of patients’ symptoms have a good effect [31].

The predominant contributor to COPD is airway inflammation, and small airway inflammation is also the main lesion site of COPD, which will lead to a large number of inflammatory cells leaching, but also release inflammatory factors IL-6, IL-8 and TNF-α, resulting in restricted airflow and eventually pulmonary fibrosis [32,33]. The increase of neutrophils is the central link of COPD, and IL-6 can suppress the apoptosis of neutrophils but enhance the proliferation of neutrophils, while the secretion of IL-8 and IL-6 is also correlated [34]. Airway inflammation can also cause thickening of airway wall and hyperplasia and hypertrophy of bronchial airway smooth muscle, leading to airway remodeling which is a hallmark pathological change of COPD [35]. Oxidative stress is a critical player in the pathogenesis of COPD [36]. The imbalance of the oxidative/antioxidant system in the body of COPD patients aggravates the nonspecific inflammatory response of the airway, resulting in the advancement of patient’s disease [37]. Several studies have also shown that long-term use of antioxidants can improve lung function, delay its decline, improve quality of life, and reduce the number of acute exacerbations in patients suffering from COPD [38,39]. SOD, MDA and TAOC are important indexes of oxidative stress in COPD. Studies have shown that COPD patients have increased plasma MDA levels, decreased SOD levels, decreased TAOC levels,, and MDA level was testified to have close relation with forced expiratory volume in one second (FEV1) [40–42]. Shenqi injection can also down-regulate the level of MDA, IL-6, sIL-2 R, TNF-α and IL-1β, and up-regulate the level of GSH-Px and SOD, and improve the oxidative stress as well as immune inflammatory response of COPD [43]. In this study, MP ameliorated in vitro COPD injury by increasing the levels of SOD and GSH-Px and decreasing the levels of MDA, TNF-α, IL-6 and IL-1β to reduce the inflammatory response and oxidative stress in CSE. -induced BEAS-2B cells. With the deepening of the research on apoptosis in the field of inflammatory diseases, people have begun to pay attention to the death mode of lung tissue in COPD patients, not only necrosis, but also apoptosis, which may play a certain role in the pathogenesis [44]. In this study, MP improved the viability and suppressed the apoptosis of BEAS-2B cells caused by CSE.

FPR2/AMPK pathway is involved in the COPD development. The expression of FPR2 in Th/Tc cells and ANXA1 (endogenous ligand of FPR2) in serum of COPD patients were lower than that of healthy nonsmokers [45]. Triterpene Acids inhibited inflammation in COPD by activating AMPK/Nrf2 and blocking NF-κB pathways [46]. FPR2 endogenous agonists promoted inflammatory biological functions in COPD [47]. The expression of AMPK was decreased in skeletal muscle tissues of COPD rats [48]. AMPK expression was suppressed in mouse lungs with emphysema and CSE-treated NHBE cells [49]. AMPK also exhibited protective effects on lung inflammatory responses and airspace enlargement [50,51]. FPR2/AMPK pathway was de-activated in BEAS-2B cells caused by CSE and MP activated the FPR2/AMPK pathway in CSE induced BEAS-2B cells, which was consistent with the above-mentioned results. In addition, knockdown of ANXA1 enhanced the inflammatory response and oxidative stress, and suppressed the FPR2/AMPK pathway of CSE-induced BEAS-2B cells treated with MP. It indicated that MP relieved the injury of BEAS-2B cells caused by CSE through promoting ANXA1 expression.

## Conclusion

Here, FPR2/AMPK pathway was found to be activated in MP treatment for CSE-induced BEAS-2B cells. In addition, MP inhibited the inflammation, apoptosis and oxidative stress of bronchial epithelial cells induced by CSE to alleviate the in vitro COPD injury by upregulating ANXA1 expression. Furthermore, knockdown of ANXA1 could reverse the effect of MP on the above changes in CSE-induced BEAS-2B cells. There are also existing some limitations. First, the application of FPR2 agonist will be better to explore the role of FPR2/AMPK pathway in CSE-induced BEAS-2B cells. Secondly, whether MP treatment affect glucocorticoid receptor (GR) and NF-κB signaling pathway activation needs to be explored in future. Thirdly, an in vivo COPD model will justify the present conclusion in our future study.

## Data Availability

The experimental data will be available on the request.
